# Mutational spectrum of the *APC* and *MUTYH* genes and genotype–phenotype correlations in Brazilian FAP, AFAP, and MAP patients

**DOI:** 10.1186/1750-1172-8-54

**Published:** 2013-04-05

**Authors:** Giovana Tardin Torrezan, Felipe Cavalcanti Carneiro da Silva, Érika Maria Monteiro Santos, Ana Cristina Victorino Krepischi, Maria Isabel Waddington Achatz, Samuel Aguiar Junior, Benedito Mauro Rossi, Dirce Maria Carraro

**Affiliations:** 1 Laboratory of Genomics and Molecular Biology, A. C. Camargo Hospital, São Paulo, SP, Brazil; 2 National Institute of Science and Technology in Oncogenomics (INCITO), São Paulo, SP, Brazil; 3 Colorectal Tumors Department, A. C. Camargo Hospital, São Paulo, SP, Brazil; 4 Laboratory of Structural Genomics, A. C. Camargo Hospital, São Paulo, SP, Brazil; 5 Oncogenetics Department, A. C. Camargo Hospital, São Paulo, SP, Brazil; 6 Barretos Cancer Hospital, Pio XII Foundation, Rua Dona Adma Jafet 74 cj. 145, São Paulo, SP, CEP: 01308-050, Brazil; 7 Laboratory of Genomics and Molecular Biology, International Center of Research - A. C. Camargo Hospital, Rua Taguá, 440, São Paulo, SP, CEP: 01508-010, Brazil

**Keywords:** *APC*, *MUTYH*, Genotype-phenotype, Mutation screening, Polyposis

## Abstract

**Background:**

Patients with multiple colorectal adenomas are currently screened for germline mutations in two genes, *APC* and *MUTYH*. *APC*-mutated patients present classic or attenuated familial adenomatous polyposis (FAP/AFAP), while patients carrying biallelic *MUTYH* mutations exhibit MUTYH-associated polyposis (MAP). The spectrum of mutations as well as the genotype-phenotype correlations in polyposis syndromes present clinical impact and can be population specific, making important to obtain genetic and clinical data from different populations.

**Methods:**

DNA sequencing of the complete coding region of the *APC* and *MUTYH* genes was performed in 23 unrelated Brazilian polyposis patients. In addition, mutation-negative patients were screened for large genomic rearrangements by multiplex ligation-dependent probe amplification, array-comparative genomic hybridization, and duplex quantitative PCR. Biallelic *MUTYH* mutations were confirmed by allele-specific PCR. Clinical data of the index cases and their affected relatives were used to assess genotype–phenotype correlations.

**Results:**

Pathogenic mutations were identified in 20 of the 23 probands (87%): 14 in the *APC* gene and six in the *MUTYH* gene; six of them (30%) were described for the first time in this series. Genotype-phenotype correlations revealed divergent results compared with those described in other studies, particularly regarding the extent of polyposis and the occurrence of desmoid tumors in families with mutations before codon 1444 (6/8 families with desmoid).

**Conclusions:**

This first comprehensive investigation of the *APC* and *MUTYH* mutation spectrum in Brazilian polyposis patients showed a high detection rate and identified novel pathogenic mutations. Notably, a significant number of *APC*-positive families were not consistent with the predicted genotype-phenotype correlations from other populations.

## Background

Patients with multiple colorectal adenomas are screened for germline mutations in two distinct genes, *APC* and *MUTYH*. According to the polyp number and age of onset, the phenotype of *APC*-mutated patients can be classified as classical familial adenomatous polyposis (FAP: more than 100 polyps, early onset) or attenuated FAP (AFAP: fewer than 100 polyps with later onset) [1-3]. *MUTYH* biallelic mutation carriers usually present 10 to 100 polyps and are categorized as having *MUTYH*-associated polyposis (MAP) [4].

FAP/AFAP (OMIM #175100) is a dominantly inherited colorectal cancer (CRC) predisposing syndrome [1,2] caused by mutations in the tumor suppressor gene adenomatous polyposis coli (*APC*). The encoded APC protein controls β-catenin turnover in the Wnt pathway [5,6]. Besides colonic polyposis and colorectal cancer, individuals with FAP can present a number of benign extracolonic features, including multiple osteomas, epidermoid cysts, desmoid tumors, and congenital hypertrophy of the retinal pigment epithelium [2]. Over 1100 different pathogenic *APC* mutations have been reported to date in the Leiden Open Variation Database (http://www.lovd.nl/2.0/), the majority of them being nonsense mutations or small insertions or deletions that lead to a truncated protein. Mutations causing AFAP have been reported to occur mainly in three regions of *APC*: at the 5^′^ end (the first five exons), in the alternatively spliced region of exon 9, or at the 3^′^ end (after codon 1580) [7-9].

MAP (OMIM #608456) is a recessively inherited syndrome caused by biallelic mutations in the mutY homolog (*MUTYH*) gene that maps to chromosome 1p34.1 [4]. *MUTYH* encodes a DNA glycosylase that plays a key role in the base excision repair pathway by removing mispaired bases caused by the oxidation product 8-oxoG [4]. Nearly 300 different sequence variants have been identified in this gene (LOVD Mutation Database), including about 80 pathogenic mutations distributed throughout the gene at positions corresponding to different functional domains of the encoded protein [10]. In contrast to *APC* pathogenic variants, which mostly result in a truncated or absent protein, most *MUTYH* pathogenic variants are missense substitutions and only a minority are splice site or truncating mutations [11].

With regard to clinical features, most *MUTYH-*mutated patients present 10 to 100 colorectal adenomas, usually with later onset compared with FAP patients [4,12,13]. *MUTYH* mutation carriers represent approximately 7.5% of patients with more than 100 adenomas without an *APC* mutation, 40% of all patients with 10–100 polyps, and 0.3–1.7% of patients with fewer than 10 polyps and early-onset CRC with no family history [12,14-16]. Furthermore, it has also been reported MAP patients having no polyps at the time of CRC diagnosis [17,18].

Because of the observed overlap between the clinical phenotype of FAP/AFAP and MAP syndromes, the identification of the causative mutation has important implications for family management, allowing effective clinical surveillance and accurate genetic counseling. Moreover, the spectrum of mutations and the genotype–phenotype correlations may have clinical impact and can be population-specific. Therefore, it is important to obtain genetic and clinical data from FAP/AFAP and MAP families in different populations.

The aim of this study was to conduct a comprehensive molecular analysis to determine the spectrum of point mutations and large genomic rearrangements (LGR) in the *APC* and *MUTYH* genes in a series of 23 Brazilian polyposis patients. This paper summarizes the mutation screening data and outlines the most cost-effective approach to detect *APC* and *MUTYH* mutations in Brazilian polyposis patients. In addition, we discuss the genotype–phenotype associations found in these families in the context of previously described data from the literature.

## Methods

### Patients

The study examined the Hereditary Colorectal Cancer Registry of A. C. Camargo Hospital (São Paulo, Brazil) [19], for families clinically suspected for FAP (> 100 colorectal adenomas) or AFAP/MAP (10–100 colorectal adenomas), enrolled between January 1998 and July 2011. Between 2010 and 2011, the genetic test was offered to forty registered unrelated polyposis families, from which 23 were available and willing to undergo genetic testing. Index patients were interviewed after providing informed consent and the family history was obtained through verbal report and, whenever possible, confirmed with clinical or pathological reports.

This study was performed in compliance with the Declaration of Helsinki and was approved by the ethics committee of A. C. Camargo Hospital (approval number: 1169/08-B). Once a mutation was identified in the index case, genetic counseling and molecular testing were offered to relatives.

### PCR and sequence analysis

Mutation screening was performed by capillary sequencing of all coding exons of the *APC* [GenBank:NM_000038.5] and *MUTYH* [GenBank:NM_001128425.1] genes, including the intron–exon boundaries. Patients clinically suspected for FAP (> 100 polyps) were first screened for *APC* mutations, while patients with attenuated polyposis (< 100 polyps) were first screened for *MUTYH* mutations. Patients negative for the first screened gene were then screened for the remaining one.

Genomic DNA was obtained from leukocytes using a Puregene Genomic DNA Isolation Kit (Gentra Systems, Minneapolis, MN, USA) according to the manufacturer’s instructions. PCR reactions used 25 ng of template and 500 nM of each primer in a final volume of 20 μl with GoTaq Green Master Mix (Promega, Madison, WI, USA). Approximately 200 ng of PCR-amplified fragments were purified with ExoSAP-IT (USB Corporation, Cleveland, OH, USA) and sequenced in both directions. Products were analyzed using an ABI 3130xl DNA sequencer (Applied Biosystems, Foster City, CA, USA) and the resulting sequences were aligned using CLCBio Genomics Workbench Software (Muehltal, Germany). The sequences of primers used for these analyses are available upon request. All mutations were confirmed in a second DNA sample. Mutations were recorded and referenced with respect to the cDNA sequence, using the nomenclature guidelines proposed by the Human Genome Sequence Variation Society (http://www.hgvs.org/mutnomen).

### Allele-specific PCR

Allele-specific PCR [20] was performed for *MUTYH* mutations to confirm the presence of two heterozygous alleles (compound heterozygosity). Primers were designed to be specific for the wild-type or mutated nucleotide of one of the *MUTYH* mutations. Sequencing of allele-specific PCR amplicons was performed to reveal the haplotype phase of the second mutation. PCR conditions and primers are available upon request.

### LGR screening

Seven patients were selected for LGR screening using multiplex ligation-dependent probe amplification (MLPA), array-comparative genomic hybridization (aCGH), and duplex quantitative PCR (qPCR): five negative for *APC* or *MUTYH* point mutations, one with a novel *APC* missense variant, and one with a monoallelic *MUTYH* mutation. All experiments were performed in duplicate.

MLPA was performed using the SALSA P043-C1 APC Probemix kit (MRC Holland, Amsterdam, The Netherlands) following the manufacturer’s protocol. PCR products were analyzed using an ABI 3130xl DNA sequencer (Applied Biosystems - Foster City, CA, USA), and gene dosage was calculated using Coffalyser V9.4 software (MRC Holland, Amsterdam, The Netherlands).

The aCGH platform used in this study was the SurePrint G3 Human CGH Microarray Kit 4 × 180 k (G4449A; Agilent Technologies, Santa Clara, CA, USA), which has an average resolution of 18 kb, with 13 and three probes located within *APC* and *MUTYH*, respectively. Briefly, samples were labeled with Cy3- or Cy5-dCTPs by random priming. Purification, hybridization, and washing were performed as recommended by the manufacturer. Data extraction was conducted using Feature Extraction software (Agilent Technologies - Santa Clara, CA, USA). Genomic Workbench software (Agilent Technologies - Santa Clara, CA, USA) was applied to identify constitutive genomic imbalances using the statistical algorithm ADM-2, with a sensitivity threshold of 6.7, and a threshold log_2_ ratio of 0.4 for duplication and −0.4 for deletion.

Genomic alterations identified by MLPA and aCGH were validated using the duplex qPCR method previously established by our group [21].

### Variant analysis

Mutations in the *APC* or *MUTYH* genes were considered deleterious if they: a) were classified as pathogenic in LOVD database; b) introduced a premature stop codon in the protein sequence (nonsense or frameshift mutation); c) occurred at donor or acceptor splice sites; or d) were whole-exon deletions or duplications. To establish the pathogenicity of one novel missense variant, web-based programs that predict the effect of an amino acid substitution were applied (SIFT, Polyphen, and MutationTaster). In addition, the frequency of this variant was assessed in 95 healthy Brazilian individuals.

### Clinical features and genotype-phenotype correlations

The following clinical and pathological data were obtained from all families from the Hereditary Colorectal Cancer Registry of A. C. Camargo Hospital [19]: number of affected individuals, age at diagnosis, number of patients with extracolonic features, and primary sites of extracolonic tumors. The extent of polyposis burden (number of adenomas) was assessed for the index cases through colonoscopy records and/or pathological report from surgical specimens. For most family members this information was unavailable. Patients and their families were grouped according to the affected gene and the index case polyposis burden into five categories: group 1, *APC*-mutated families with fewer than 100 colorectal adenomas (attenuated polyposis); group 2, *APC*-mutated families with 100–1000 adenomas (intermediate polyposis); group 3, *APC*-mutated families with more than 1000 adenomas (severe polyposis); group 4, *MUTYH*-mutated families; and group 5, mutation-negative families.

Genotype–phenotype correlations in the three *APC-*mutated groups were compared with those previously described, as reviewed by Nieuwenhuis and Vasen (2007) [22]. This review evaluated a large number of studies in FAP patients and proposed a categorization of the phenotypes according to the severity of the polyposis and the associated site of the *APC* mutation.

Statistical evaluation was performed using the Student’s *t*-test using Prism 5 software (GraphPad, San Diego, CA, USA). Statistical significance was set at a *p*-value < 0.05.

## Results

Twenty-three Brazilian families with a clinical diagnosis of classical or attenuated polyposis were included in this study. The majority of the index cases (15) presented an intermediate or severe FAP phenotype (> 100 polyps) and 13 of them harbored an *APC* pathogenic mutation, while one patient was mutation-negative and one had a monoallelic *MUTYH* mutation. The remaining eight patients presented an attenuated polyposis burden (< 100 polyps), among whom five carried biallelic mutations in the *MUTYH* gene, one carried a novel *APC* duplication of exons 1–3, one presented a novel *APC* missense variant, and one was mutation-negative. Seven novel germline mutations (six pathogenic and one variant of unknown significance) were detected in this cohort, and two of them have been recently published by our group [21,23]. The *APC* and *MUTYH* mutation spectrum, including information about previous reports of the detected mutations, is summarized in Table 1.

**Table 1:** ***APC*and*MUTYH*mutation spectrum in Brazilian polyposis patients**

**ID**	**Gene**	**Mutation**	**Exon**	**Type**	**Ref.**^**a**^**reported N times**^**b**^	
Mutation-positive patients with > 100 polyps						
02	*APC*	del 5q21.3-q22.3 (chr5:107916475–113079330 Hg19)	1-15	gene deletion	Torrezan et al. [21]	1
04	*APC*	c.856_859dupCATG (p.Glu287Alafs*2)	8	duplication	Current study	0
05	*APC*	c.447dupC (p.Lys150Glnfs*18)	4	duplication	Current study	0
23	*APC*	c.4097dupC (p.Gln1367Serfs*8)	15	duplication	Current study	0
01	*APC*	c.904C > T (p.Arg302*)	8	nonsense	Mandl et al. [24]	22
03	*APC*	c.4348C > T (p.Arg1450*)	15	nonsense	Miyaki et al. [25]	40
06	*APC*	c.3880-3881delCA (p.Gln1294Glyfs*6)	15	deletion	Miyaki et al. [25]	1
07	*APC*	c.847C > T (p.Arg283*)	8	nonsense	Mandl et al. [24]	49
08	*APC*	c.4099C > T (p.Gln1367*)	15	nonsense	Friedl and Aretz [26]	8
10	*APC*	c.3050-3053delATGA (p.Asn1017Metfs*4)	15	deletion	Vandrovcová et al. [27]	2
11	*APC*	c.3927-3931delAAAGA (p.Glu1309Aspfs*4)	15	deletion	Miyoshi et al. [28]	304
14	*APC*	c.4393-4394delAG (p.Ser1465Trpfs*3)	15	deletion	Miyoshi et al. [28]	40
21	*APC*	c.3927-3931delAAAGA (p.Glu1309Aspfs*4)	15	deletion	Miyoshi et al. [28]	304
24	*MUTYH*	c.[1187G > C];[=] (p.[Gln396Asp];[=])	13	missense	Al-Tassan et al. [4]	532
Mutation-positive patients with < 100 polyps						
15	*MUTYH*	c.[348 + 33_*64 + 146del4285insTA]; [348 + 33_*64 + 146del4285insTA]	4-16	large deletion	Torrezan et al. [23]	2
13	*APC*	c.5365G > C (p.Val1789Leu)^c^	15	missense	Current study	0
17	*APC*	Exon 1–3 duplication	1-3	large duplication	Current study	0
16	*MUTYH*	c.[536A > G]; [1147delC] (p.[Tyr179Cys]; [Ala385Profs*23])	7;12	missense;	Al-Tassan et al. [4]	532;
				deletion	Eliason et al. [29]	71
18	*MUTYH*	c.[389-1G > C]; [536A > G] (p.[Val130GlufsX98;p.(spl?)]; [Tyr179Cys])	i4;7	splice site;	Olschwang et al. [30]	2;
				missense	Al-Tassan et al. [4]	532
19	*MUTYH*	c.[721C > T]; [721C > T] (p.[Arg241Trp]; [Arg241Trp])	9	missense	Fleischmann et al. [31]	11
447	*MUTYH*	c.[536A > G]; [1227-1228dup] (p.[Tyr179Cys]; [Glu410Glyfs*43])	7;13	missense;	Al-Tassan et al. [4];	506;
				duplication	Baglioni et al. [32]	31

^a^ The following databases were consulted: LOVD: http://www.lovd.nl/2.0/; dbSNP: http://www.ncbi.nlm.nih.gov/projects/SNP/; and 1000 Genomes: http://www.1000genomes.org/; ^b^ Number of times reported at LOVD database; ^c^ Variant of unknown clinical significance.

### *APC* mutations

Fourteen pathogenic *APC* mutations were identified in this series: three small duplications, five small deletions, four nonsense mutations, one multiple exon duplication, and one whole-gene deletion. Six of them were novel mutations (Table 1). All patients presented distinct mutations, except for two unrelated probands that presented the hotspot mutation at codon 1309 (c.3927–3931delAAAGA; p.Glu1309Aspfs*4).

One patient (ID13) presented a novel missense *APC* variant of unknown significance: c.5365G > C (p.Val1789Leu). This patient was diagnosed with attenuated polyposis at the age of 56 years, presenting around 20 polyps at the time of clinical diagnosis. *In silico* studies using three different functional prediction programs (Polyphen, SIFT and MutationTaster), which all classified the p.Val1789Leu variant as having minimal or no effect on protein function, with the following scores: 0 (Polyphen); 0.30 (SIFT); 0.87 (*P*: 0.99, MutationTaster). Because the proband was the only affected member of the family, it was not possible to perform co-segregation analysis of the variant with the disease within the family; nevertheless, this variant was not detected in a control population of 95 healthy individuals.

**Figure 1:**
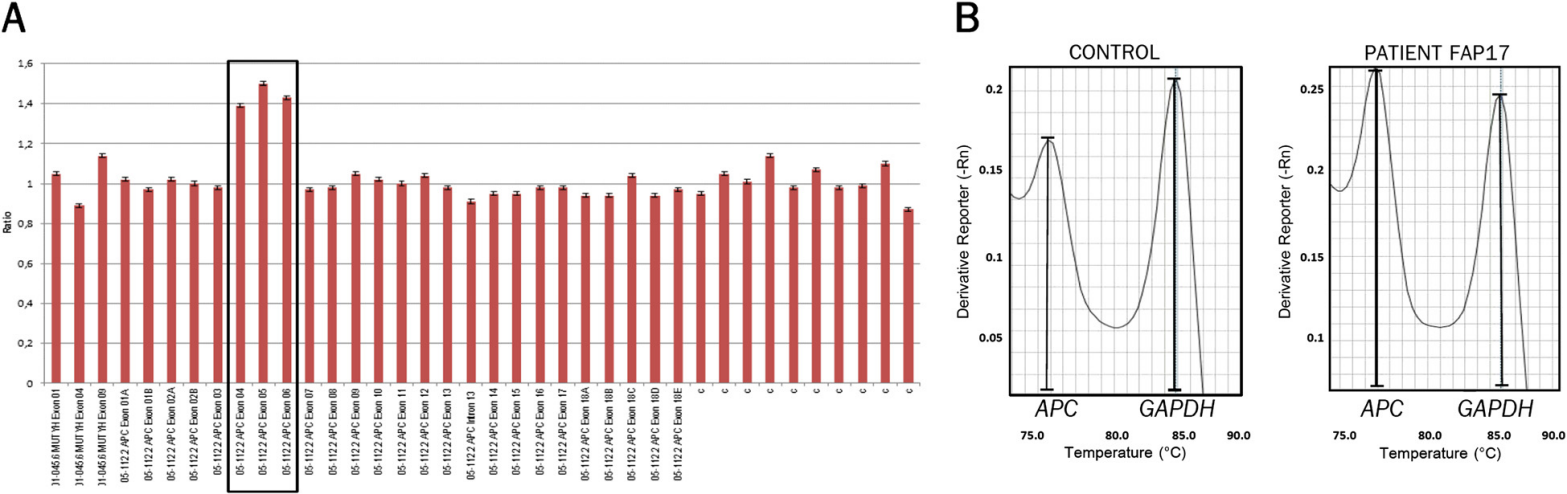
**Duplication encompassing exons 1 to 3 of the *APC *gene (patient ID17). A**: MLPA graphic showing normalized ratios of probes ordered by genomic position; the box marks the probes that indicate duplication of *APC* exons 1, 2, and 3 (exons 4, 5, and 6 according to MLPA exon numbering [GenBank:NG_008481]). **B**: Melt curve of duplex qPCR of *APC* exon 2 and *GAPDH* intron 7 (reference gene). The ratio of *APC*/*GAPDH* peaks of the melting curve was 0.72 in the control sample and 1.06 in patient ID17, leading to a normalized ratio of 1.47 for the FAP patient, which confirms the duplication.

In this series, two patients presented *APC* LGRs, identified by MLPA and/or aCGH and confirmed by gene dosage qPCR. Patient ID02 presented a 5.2-Mb deletion at 5q21.3–q22.3 that encompassed the entire *APC* gene and 19 additional genes, which have been previously published by our group [21]. The second patient (ID17) presented a duplication of *APC* exons 1–3 that was identified by MLPA (Figure 1A) and validated by duplex qPCR [21] (Figure 1B).

### *MUTYH* mutations

Biallelic germline mutations in the *MUTYH* gene were identified in five patients, among whom two were homozygotes for the causative mutation and the remaining three were compound heterozygotes for two distinct pathogenic variants. A monoallelic mutation was identified in one patient.

One patient (ID19) and her brother were homozygous for the p.Arg241Trp missense mutation, because of a consanguineous marriage between the parents. The second homozygous patient presented a deletion of exons 4–16 (c.348 + 33_*64 + 146del4285insTA) on both alleles, and stated having no known inbreeding in her family. This 4,285 bp deletion was the first LGR to be described in *MUTYH*, recently published by us [23] and by an independent group that found this deletion in a French patient [33].

For the three patients harboring two distinct pathogenic variants (ID16, ID18, and ID447), we used allele-specific PCR to confirm the biallelic nature of the mutations. All cases presented the hotspot missense mutation p.Tyr179Cys in one allele accompanied by a second truncating mutation in the remaining allele (one deletion, one duplication, and one splice site mutation).

One patient (ID24) was a monoallelic carrier of the hotspot missense mutation p.Gly396Asp, and no other mutation could be identified.

### Clinical features

Clinical records and verbal reports obtained from the 23 index patients and their relatives revealed 113 affected individuals among all families; their summarized clinical data are described in Table 2.

**Table 2:** Clinical features of the 23 Brazilian polyposis families

				**Age of onset**		**Extracolonic Manifestations**					
**Family ID**	**Affected codon**	**Polyposis burden**^**a**^	**N**^**b**^	**Range**	**Mean**	**Gastric polyps**	**Duodenal polyps**	**Desmoid tumor(n)**	**Osteoma**	**Epidermoid cysts/lipoma**	**Other tumor sites**
**1 - Attenuated FAP**	***APC***			35-56	46.3						
ID13	1789^c^	attenuated	1	56	56	yes	yes	no	no	NA	Uterus
ID17	Exon 1–3 dup	attenuated	2	35-48	41.5	yes	No	no	no	NA	Lung
**2 - Intermediate FAP**	***APC***			18-67	35.7						
ID02	Exon 1–15 del	intermediate	5	40-44	42	yes	yes	no	no	NA	
ID03	1450	intermediate	7	21-54	34.2	yes	yes	yes (2)	no	NA	Liver; Hematologic
ID04	287	intermediate	14	18-44	29.6	no	no	no	no	NA	Bilateral breast; Stomach; BCC
ID05	150	intermediate	18	29-67	40	yes	yes	yes (3)	no	yes	Breast; Melanoma
**3 - Severe FAP**	***APC***			7-58	29.2						
ID01	302	profuse	5	14-27	23.6	yes	no	no	no	NA	Brain
ID06	1294	profuse	11	20-35	27.7	yes	yes	yes (1)	yes	yes	
ID07	283	profuse	8	27-58	37	yes	no	yes (2)	yes	yes	Liver
ID08	1367	profuse	3	15-55	35	yes	yes	yes (1)	yes	NA	
ID10	1017	profuse	15	7-55	28.1	no	no	yes (4)	yes	yes	Thyroid
ID11	1309	profuse	3	30-40	35	yes	yes	no	yes	NA	
ID14	1465	profuse	8	17-36	26.5	no	no	yes (2)	no	yes	
ID21	1309	profuse	1	18	18	yes	yes	no	yes	no	
ID23	1367	profuse	1	22	22	NA	NA	yes(1)	NA	NA	
**4 - MAP**	***MUTYH***			27-53	37.9						
ID15	Exon 4–16 del	attenuated	2	42-44	43	no	no	no	no	NA	
ID16	179; 385	attenuated	2	30-53	41.5	yes	no	no	no	NA	Uterus
ID18	130; 179	attenuated	1	45	45	no	no	no	no	NA	
ID19	241; 241	attenuated	2	27-31	29	no	no	no	no	NA	
ID24	396;[=]	intermediate	1	55	55	NA	NA	no	NA	NA	
ID447	179; 410	attenuated	2	34-35	34.5	no	no	no	no	no	
**5 - No Mutation**				26-29	27.5						
ID20		attenuated	1	26	26	no	no	no	yes	no	
ID22		intermediate	1	29	29	no	no	no	no	no	

^a^ Polyposis burden of the index case. The remaining columns are based on all affective relatives with available clinical information; ^b^ N = number of clinically affected family members; BCC = basal cell carcinoma; NA = data not available; ^c^ Variant of unknown clinical significance.

### Polyposis/CRC age at diagnosis

**Figure 2:**
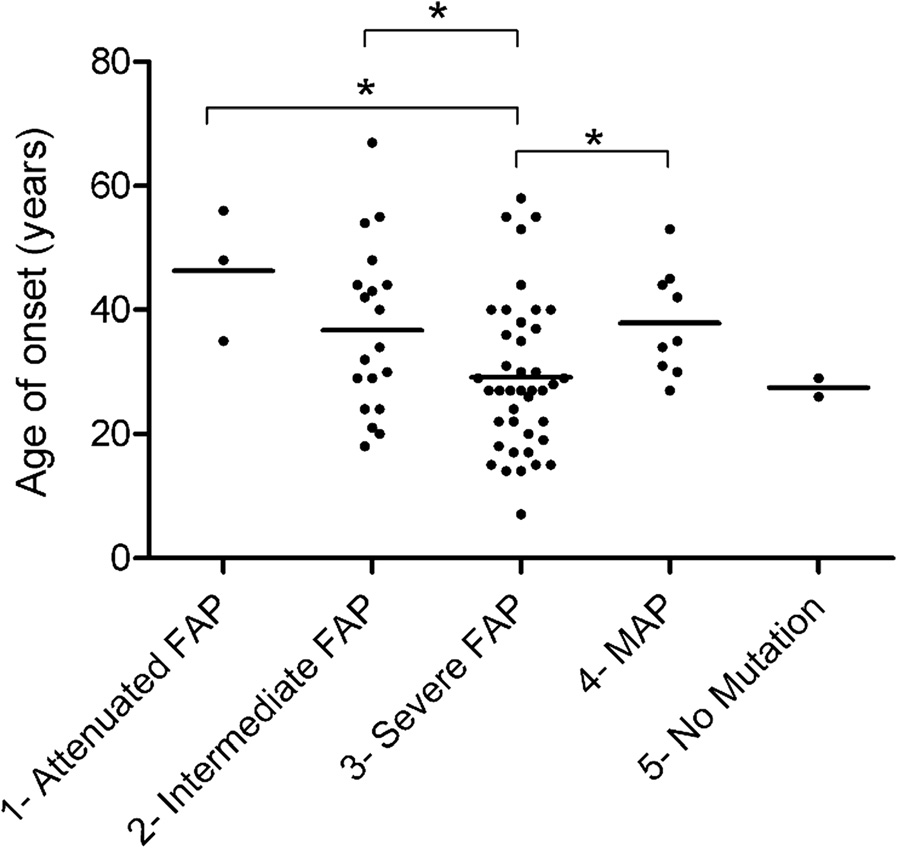
**Age of onset per group.** The graph shows the distribution and the mean (horizontal line) age of onset for each of the five defined groups. Groups 1, 2, and 4 patients had a significantly later age of onset than group 3 patients (*t* = 2.35 *p* = 0.024; *t* = 2.15 *p* = 0.04; *t* = 2.01 *p* = 0.05, respectively).

Across the entire series, the average age at diagnosis and first symptoms of CRC and/or polyposis was 32.6 years (range 7–67 years). The mean age of onset in *APC*-positive families was 46.3 years (range 35–56) for group 1 (attenuated FAP); 35.7 (range 18–67) for group 2 (intermediate FAP); and 29.2 (range 7–58) for group 3 (severe FAP). MAP families presented a mean age of 37.9 years (range 27–53 years); while the average age of onset in families with no identified mutation was 27.5 years (range 26–29 years). Comparison among the five groups revealed that the *APC*-positive group 1, group 2, and MAP patients demonstrated a later age of onset compared with the severe FAP patients (group 3) (Figure 2).

### Extracolonic manifestations

Extracolonic manifestations were reported in all *APC*-mutated families (Table 2). Gastric and duodenal polyps (upper gastrointestinal polyps) were the most common extracolonic manifestations observed in these patients, and occurred in 11/14 families (79%) and across all three *APC* groups. Osteomas were observed most often in the severe FAP patients (6/9), and epidermoid cysts/lipomas occurred in the intermediate and severe FAP patients (5/13).

Desmoid tumors were observed in 8/14 *APC*-positive families (57%) and were associated with different mutation sites, with only two of them occurring after codon 1444. Five families had more than one individual affected by desmoid tumors.

MAP families had fewer extracolonic manifestations: only one family presented upper gastrointestinal polyps and none presented desmoid tumors.

Regarding other tumor sites, papillary thyroid carcinoma appeared in one family of *APC* group 3; liver and breast cancers were reported in two families each: one from group 2 and one from group 3; uterine cancer was reported in one group 1 and one group 4 (MAP) family. Finally, lung, hematologic, brain, or skin cancer and melanoma were reported in one family each.

### Comparison with described *APC* genotype–phenotype correlations

**Figure 3:**
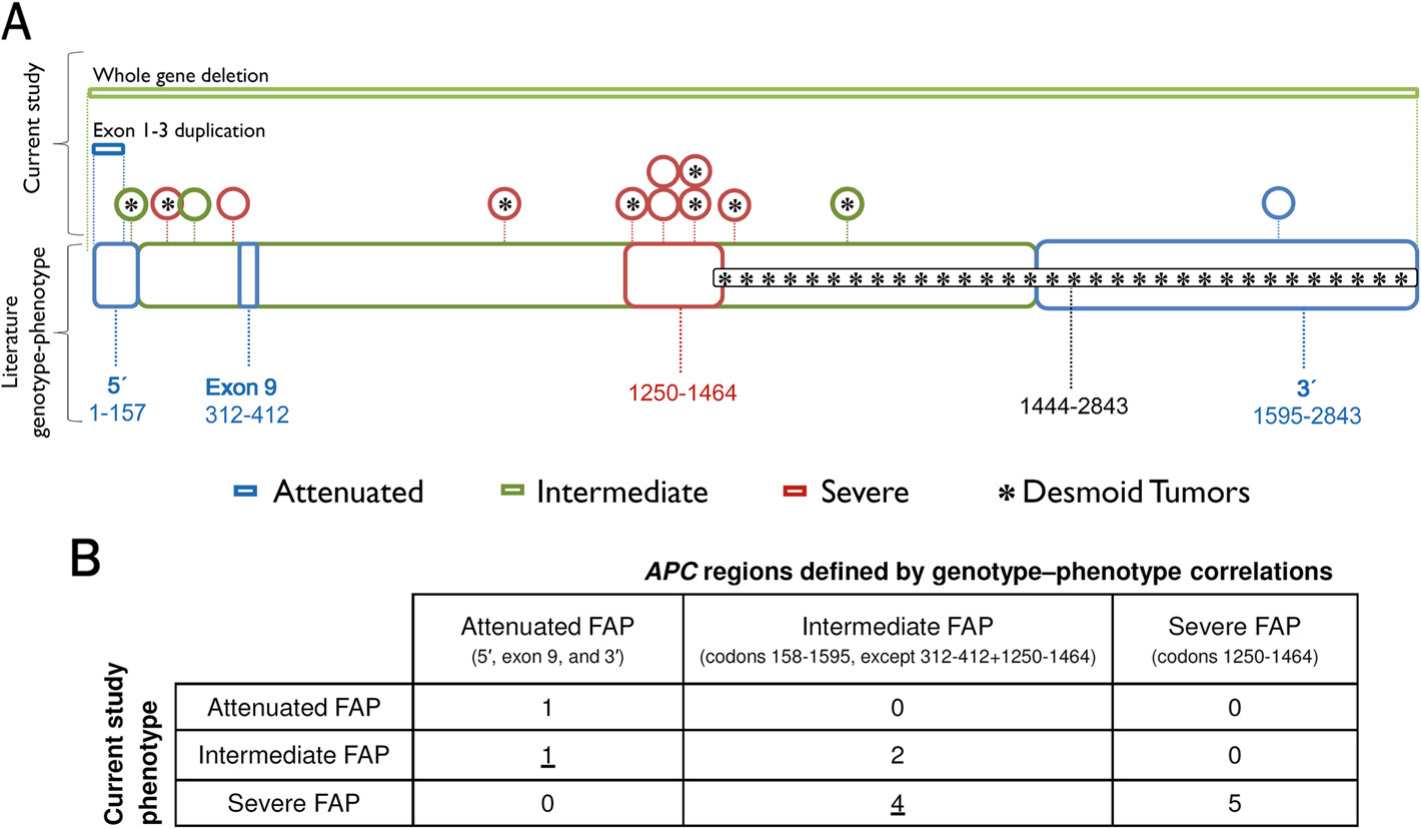
**Genotype–phenotype correlation. A**: Distribution of the polyposis phenotype of the index case and the presence of desmoid tumors along the *APC* gene. Schematic representation of the two large genomic rearrangements (top bars) and the 13 point mutations or small insertions/deletions (circles) identified in this series (including the variant of unknown significance – last circle). The asterisk inside the circles denotes patients with desmoid tumors. The lower, thick bar represents the *APC* regions defined by genotype–phenotype correlations proposed by Nieuwenhuis and Vasen (2007) [22]. Genotype–phenotype correspondence between our results and those previously published is indicated by concordant colors (blue/green/red/black). Numbers represent *APC* codons. **B**: Number of families presenting *APC* point mutations (N = 13) according to the index case polyposis phenotype and the *APC* codon limits, showing that five individuals (underlined numbers) presented a polyposis burden different from that predicted.

Genotype–phenotype correlations in polyposis syndromes have been evaluated in several studies, and a general association between the location of the mutation and the clinical manifestation has been observed, albeit with some inconsistencies [7,21,34-36]. Recently, Nieuwenhuis and Vasen (2007) [22] performed a meta-analysis and proposed categorization of the phenotypes into three degrees of polyposis severity and the associated site of *APC* mutation. Attenuated FAP was associated with mutations before codon 157, after codon 1595, and in the alternatively spliced region of exon 9; severe polyposis was related to mutations between codons 1250 and 1464; and an intermediate phenotype was associated with *APC* mutations located in the remaining sequence of the gene (Figure 3).

We compared the clinical and genetic data from our cohort with the *APC* codon limits defined by Nieuwenhuis and Vasen (2007) [22]. Figure 3A and B show the distribution of the polyposis phenotype of the index cases according to the location of their *APC* mutation, and compare it with the genotype–phenotype correlations previously proposed. Nine (64%) of 14 FAP families with an *APC* pathogenic mutation presented the expected polyposis severity according to the location of the *APC* mutation, while the remaining five families exhibited discordant results from the anticipated phenotype. Nine families presented a profuse polyposis burden (> 1000 polyps) — two of them carried a mutation in codon 1309, two in codon 1367, and one in codon 1294, while the four remaining patients carried mutations in codons usually associated with an intermediate number of polyps (codons 283, 302, 1017, and 1465) (Figure 3A and B).

### Particular phenotypes

Two of the herein identified mutations presented a remarkably more aggressive phenotype than expected given their location and the phenotypes reported in the literature. The mutation c.447dupC (p.Lys150GlnfsX18), identified in family ID05, is located in exon 4; the 5^′^ region of the *APC* gene is generally associated with attenuated polyposis and later age of onset. While the mean age of onset in this family (40 years old) was within the predicted range, the polyposis phenotype caused by this novel mutation was more aggressive than expected, since several members of the family presented more than 100 adenomatous polyps. Furthermore, three relatives had developed a desmoid tumor, usually not observed in patients with a mutation at the 5^′^ end of the *APC* gene.

**Figure 4:**
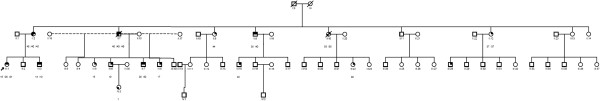
**Family tree of ID10 family.** This family harbored a truncating mutation at codon 1017 located in a region usually associated with an intermediate FAP phenotype. This mutation displayed an aggressive phenotypic expression: the proband (individual III:1, indicated by an arrow) presented her first polyposis symptoms at the age of 15, a desmoid tumor at age 20, and a thyroid carcinoma at age 21. When available, the ages of onset are presented under each individual. Her brother (III:2) and six first cousins (III:5, 6, 7, 8, 15, and 22) also developed polyps at early ages (14 to 29 years old). The most prematurely affected was a second cousin (individual IV:3), who was diagnosed with polyps at the age of 7. Desmoid tumors were described in another three relatives: two uncles at the age of 40 (II:7 and II:9) and one cousin at 32 years old (III:7).

An aggressive phenotypic expression was also observed for the mutation c.3050–3053delATGA (p.Asn1017MetfsX4), identified in family ID10 (Figure 4). Although the mutation is located in the region associated with an intermediate FAP phenotype, the proband presented a high number of polyps (> 1000) at the age of 15, a desmoid tumor at the age of 20, and thyroid carcinoma and jaw keratocysts at 21 years. Her brother and seven cousins also developed polyps at early ages (7, 14, 15, 19, 17, 22, and two at 29 years old). Desmoid tumors were described in another three relatives.

## Discussion

This is the first report of a comprehensive mutational analysis and genotype–phenotype correlation in Brazilian polyposis families. Through direct sequencing of the *APC* and *MUTYH* genes, MLPA, aCGH, and duplex qPCR, we were able to identify pathogenic mutations in 20 of 23 index cases — a detection rate of 87%. Of the remaining three patients, two were mutation-negative and one harbored a novel *APC* missense variant (p.Val1789Leu). Because of the lack of affected relatives for co-segregation analysis and the inconclusive results given by *in silico* analysis and control population screening, the clinical significance of this alteration is yet to be determined. Interestingly, a parallel study from our institution, performed in high risk cancer patients, revealed that this patient also presents a rare germline microdeletion of the *PIP* gene possibly associated with an increased cancer risk (Silva 2013, unpublished observations), suggesting that these two alterations may be acting in synergy.

The detection rate in polyposis patients from other populations varies markedly, ranging from 39 to 90%; the variation reflects different selection criteria for testing and diverse sensitivity of screening strategies [4,27,37-40]. Our data reinforce the need to apply a combination of mutation-screening methods to detect the disease-causing mutation in polyposis patients efficiently. Most *APC* mutations previously described were identified with conventional methods, for instance denaturing high performance liquid chromatography or the protein truncation test, which can have a relatively low detection rate. Nowadays, the gold standard detection method for polyposis patients is direct DNA sequencing of all *APC* and *MUTYH* coding exons (including intron–exon boundaries), accompanied by screening for LGR, as was performed here.

The majority of mutations identified in our cohort were distinct, except for two families who shared the codon 1309 hotspot *APC* mutation and three families who presented the p.Tyr179Cys hotspot mutation in one of the *MUTYH* alleles. The absence of the commonly reported *APC* mutation at codon 1061, and the relatively low frequency of the hotspot mutations *APC* 1309, *MUTYH* p.Tyr179Cys, and p.Gly396Asp are consistent with the fact that Brazilian patients represent an admixed population, probably lacking a founder *APC* or *MUTYH* mutation.

The p.Tyr179Cys and p.Gly396Asp *MUTYH* mutations were identified in three families and one family, respectively, and corresponded to 44% of all mutated alleles identified in this gene (4/9). These are also the most prevalent mutations in populations of European origin, probably because of a founder effect, and account for approximately 80% of all reported mutant alleles [12]. A recent report described a screen for these two variants in 30 Brazilian patients with clinical phenotypes of MAP and FAP; 5/30 patients were identified as carrying one of these two hotspot mutations, and four of them were in a biallelic state [41]. However, because the entire coding sequence of *MUTYH* was not evaluated in all patients in this study, we cannot perform comparisons with the frequency found in our patients.

In our series, we could not identify a causative mutation in two index cases—one with attenuated polyposis and one with an intermediate polyposis phenotype. A possible explanation for the polyposis phenotype in these patients is the presence of unusual mutations in the *APC* or *MUTYH* genes, such as intron or promoter point mutations, epimutations or genetic mosaicism. In this sense, a recent study demonstrated that up to 8% of *APC/MUTYH*-negative polyposis patients presented a deep intronic *APC* variant that led to an aberrant transcript [42]. A second possibility is the existence of other susceptibility genes, and with the current possibility of screening all coding genes by next generation exome sequencing, it can be anticipated that novel polyposis-predisposing genes will be identified.

Considering that this study is the first comprehensive analysis of *APC* and *MUTYH* mutations in Brazilian polyposis patients, we attempted to determine the most cost-effective approach to detect the causative mutation in this population. In patients presenting fewer than 100 polyps (N = 8), 62% carried a biallelic mutation in the *MUTYH* gene. Among patients with more than 100 polyps (15 cases), three cases presented a mutation in *APC* exon 8 and eight cases (53%) exhibited a mutation between codons 1017 and 1650 of *APC* exon 15. Interestingly, this initial region of exon 15 comprises only 16% of the coding sequence of *APC,* yet presented a high mutation rate in our cohort. Therefore, based on our results, an optimized scheme for the molecular diagnosis of *APC* and *MUTYH* mutations in the Brazilian population might be obtained as follows: for patients presenting > 100 polyps, codons 1017–1650 of *APC* exon 15 should be sequenced first, followed by exon 8, and then the remaining *APC* exons; for patients presenting fewer than 100 polyps, *MUTYH* should be firstly screened*.* Because none of the studied *MUTYH* patients presented only the hotspot mutations p.Tyr179Cys or p.Gly396Asp, the whole gene should be sequenced, instead of undertaking an initial search for these variants, as recommended for other populations [18].

Genotype–phenotype correlations in polyposis syndromes are of great clinical interest, because they can contribute to better genetic counseling and simplify mutation screening. In several studies, an association between the location of the mutation and the clinical manifestations has been observed [7,20,27,34-36]. However, since several contradictions have also been reported [22], it remains unclear whether the genetic information should guide clinical decision-making, such as the extent of the prophylactic colectomy or the protocol for clinical surveillance [7,43-47].

Regarding the age at which clinical surveillance should begin, the established guidelines suggest that classical FAP patients should start endoscopic surveillance from the early teens, while AFAP and MAP families could start surveillance at age 18–20 [46]. Similar to the literature, our results demonstrated that severe FAP patients had an earlier age of onset (on average 10 years younger than AFAP or MAP patients). However, the most premature case in our series was a 7-year-old patient from a family with an *APC* mutation at codon 1017 — a region usually associated with an intermediate phenotype. A particularly aggressive phenotypic expression of this mutation was observed in this family; several relatives presented a high number of polyps (> 1000), an early age of onset, and desmoid tumors. This case demonstrates the importance of considering the family clinical history when planning the surveillance of other family members.

One of the most clinically important discrepancies observed in our study concerns desmoid tumors, which, even if histologically benign, can lead to life-threatening complications through their size and impingement on vital structures. Indeed, desmoid tumors represent the second leading cause of death in FAP patients [46,48] and were identified at high frequency in our cohort, occurring in 57% (8/14) of *APC*-mutated families. Although described as usually associated with mutations after codon 1444 [22], only two out of eight families affected by desmoid tumors in our study harbored mutations after this codon. In concordance with our findings, previous studies with large cohorts have also failed to confirm this association [49], or described different boundaries for the increased risk of these tumors, such as codon 1310 [50] or 1395 [35]. Furthermore, besides the location of *APC* mutations, several other risk factors are suggested to be related with desmoid tumor development, such as surgical trauma [51], pregnancy [52] and especially positive family history for desmoid tumors [49]. In this regard, the last appears to be the most important risk factor for our population, since five of eight families with desmoid tumors presented more than one relative affected by this tumor.

The differences observed in certain phenotypic features between our series and those of others may be because of our relatively small number of FAP and MAP families, may reflect some selection or data collection bias, or may be related to phenotypic peculiarities in this specific population. In this sense, the majority of FAP genotype-phenotype studies were performed in Europeans cohorts [34-40,43,47,49] and the self-declared ethnic origin of most families from our study was also European (Portuguese, Italian and Spanish, mainly) - except from two Japanese and one Arabian families. However, it is important to highlight that Brazilians represent an extremely admixed population, with most individuals presenting some degree of African and Amerindian ancestry [53].

Furthermore, intra and interfamilial variations in the FAP phenotype are also well documented in other populations, and it is likely that modifying genes and environmental factors, as well as functional polymorphisms of the normal *APC* allele, play a crucial role in determining the clinical course of disease [54,55]. In this regard, the different genetic background and/or environmental factors of our population could be responsible for the phenotypic differences observed in our study. For instance, in our series, desmoid tumors were much more prevalent in *APC*-mutated patients (57%) than in others previous studies (10-15%) [22,49,54], indicating that perhaps our set of patients represents a distinct group regarding this extracolonic feature.

Finally, the lack of a clear phenotypic expression of the mutations identified in our study complicates clinical predictions based on knowledge of the mutation site, and as a result, we can make no specific surveillance and management recommendations for the Brazilian population. In order to accomplish that, larger studies need to be carried out. Instead, we recommend that clinical decisions regarding an individual patient should not be based strictly on the genotype, but mainly on the colonic phenotype and family clinical history.

## Conclusions

In this comprehensive investigation of the *APC* and *MUTYH* mutation spectrum in Brazilian polyposis patients, we identified a high frequency of germline mutations, allowing the identification of several novel pathogenic variants and the proposal of a cost-effective screening approach for this population. Notably, a significant number of *APC* mutation-positive families were not consistent with predicted genotype–phenotype correlations, and this should be taken into consideration for genetic counseling and patient management of our population.

## Competing interests

The authors declare that they have no competing interests.

## Authors’ contributions

GTT, DMC, and BMR conceived and designed experiments. GTT, FCCS, ACVK, and DMC performed and analyzed experiments. EMMS, MIA, SAJ, and BMR assessed clinical data and selected patients. BMR and DMC contributed reagents/materials/analysis tools. GTT, FCCS, ACVK, BMR, and DMC wrote/edited the paper. All authors have read and approved the final manuscript.
